# Patients with (familial) atrial fibrillation: take off the sweater

**DOI:** 10.1007/s12471-024-01890-8

**Published:** 2024-08-19

**Authors:** Andrea Bochem, Lucas V. A. Boersma, Saskia N. van der Crabben

**Affiliations:** 1 Department of Cardiology, St. Antonius Ziekenhuis, Nieuwegein, The Netherlands; 2 Department of Human Genetics, Amsterdam University Medical Centre, Amsterdam, The Netherlands

A 25-year-old man was referred for genetic analysis of his cardiac arrhythmia. The otherwise healthy man had experienced exercise-induced atrial fibrillation (AF) at the age of 16 years. Seven years later AF recurred, again induced by exercise. He underwent chemical conversion. The findings of an extended cardiological examination (cardiac MRI and exercise test) were normal, revealing only a sinus bradycardia of 52 beats/min. His maternal grandfather had AF and received a pacemaker at the age of 79 years because of symptomatic bradycardia. During history taking at the clinical genetics outpatient clinic the skin abnormality shown in Fig. 1 was noted. The patient reported that his paternal grandfather had the same skin abnormality. What is the diagnosis of this skin abnormality and (how) does it relate to his (familial) AF?

**Fig. 1:**
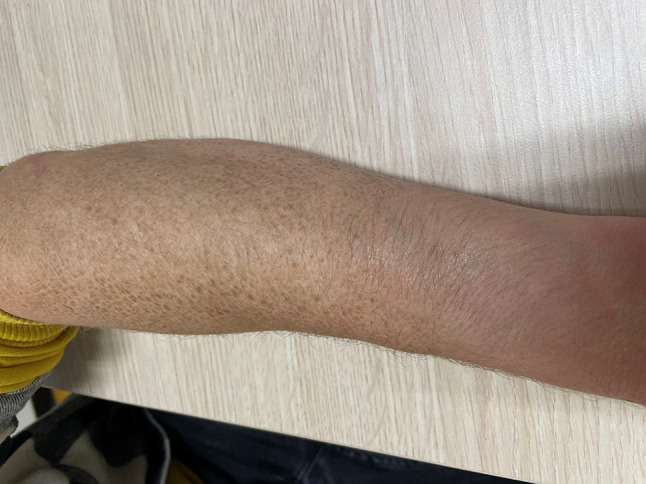
Forearm of the patient (with patient’s oral and written permission)

## Answer

You will find the answer elsewhere in this issue.

